# Weighing the risks of high intakes of selected micronutrients compared with the risks of deficiencies

**DOI:** 10.1111/nyas.14128

**Published:** 2019-06-06

**Authors:** Reina Engle‐Stone, Stephen A. Vosti, Hanqi Luo, Justin Kagin, Ann Tarini, Katherine P. Adams, Caitlin French, Kenneth H. Brown

**Affiliations:** ^1^ Department of Nutrition University of California Davis California; ^2^ Department of Agricultural and Resource Economics University of California Davis California; ^3^ Kagin's Consulting Vacaville California; ^4^ Independent consultant Laval Quebec Canada

**Keywords:** micronutrient, tolerable upper intake level, dietary intake

## Abstract

Several intervention strategies are available to reduce micronutrient deficiencies, but uncoordinated implementation of multiple interventions may result in excessive intakes. We reviewed relevant data collection instruments and available information on excessive intakes for selected micronutrients and considered possible approaches for weighing competing risks of intake above tolerable upper intake levels (ULs) versus insufficient intakes at the population level. In general, population‐based surveys in low‐ and middle‐income countries suggest that dietary intakes greater than the UL are uncommon, but simulations indicate that fortification and supplementation programs could lead to high intakes under certain scenarios. The risk of excessive intakes can be reduced by considering baseline information on dietary intakes and voluntary supplement use and continuously monitoring program coverage. We describe a framework for comparing risks of micronutrient deficiency and excess, recognizing that critical information for judging these risks is often unavailable. We recommend (1) assessing total dietary intakes and nutritional status; (2) incorporating rapid screening tools for routine monitoring and surveillance; (3) addressing critical research needs, including evaluations of the current ULs, improving biomarkers of excess, and developing methods for predicting and comparing risks and benefits; and (4) ensuring that relevant information is used in decision‐making processes.

## Introduction

Deficiencies of micronutrients (MNs), such as iron and vitamin A (VA), have been recognized for decades as a public health problem disproportionately affecting women and young children in low‐ and middle‐income countries (LMICs) and resulting in unnecessary morbidity and mortality.^1^, ^2^ In response, governments and national and international organizations have implemented programs to address these deficiencies, including large‐scale food fortification, biofortification, MN supplementation, and behavior change communication aimed at changing food production decisions and consumer practices related to food selection and preparation. These diverse programs are typically planned, managed, and supported by different national and international stakeholders, often leading to a lack of coordination among these efforts.^3^ In addition, monitoring systems are often weak or nonexistent, so information is not always available on program coverage or quality of implementation.^4^, ^5^ Many programs occur in the contexts of changing dietary habits that include increases in the consumption of processed foods, some of which are fortified, and self‐administered vitamin and mineral supplements. This situation has led to new concerns about the possibility of excessive MN intakes imposed by these programs.^6^, ^7^, ^8^ It is therefore necessary to develop systems for monitoring program reach and population nutritional status to understand the extent and severity of any risks associated with excessive MN intakes, and to use this information to manage programs effectively and efficiently to improve MN nutrition safely.

The objectives of this paper are to: (1) review existing measures of the impact of nutrition interventions (in terms of both benefits and risks) and existing instruments used to assess the amounts of vitamins and minerals being dispensed by all possible simultaneous ongoing interventions in a country or region, in addition to usual dietary intakes; (2) assess the burden of excessive intakes according to available literature and present simulated benefit and risk results for selected MN programs, using Cameroon as a case study; (3) discuss frameworks for assessing the potential trade‐offs between increases in program reach to individuals at risk of deficiency and risk of excessive MN intake; and (4) provide input on what program indicators and mechanisms for data collection may be useful for monitoring the risk of excessive intake of vitamins and minerals delivered through public health interventions. We focus primarily on VA, folic acid, iron, and zinc intakes in LMICs. Since there is relatively little information on the potential for excessive intakes in LMICs, we include selected data from high‐income settings, where this information may be useful to draw inferences about the current or future situations for some populations in LMICs, particularly in light of rapidly changing food environments around the world.

## Indicators for measuring micronutrient program impact and safety

To manage the benefits and risks of nutrition programs, information is needed on (1) the distribution of inadequate and excessive intakes, (2) the population subgroups most likely to be affected, and (3) the relative contributions of different nutrient sources (including the usual diet, self‐administration of supplements, and the types and performances of existing and alternative intervention programs). Possible measures of program impact are summarized in Table 1, along with examples of their use.

**Table 1:** Selected measures for assessing or predicting impact of micronutrient interventions

Type of measure	Examples
Functional outcomes	Mortality
	Morbidity (e.g., incidence of diarrheal disease)
	Cognitive development
	Stunting
	Pregnancy outcomes (e.g., preterm birth)
	Birth defects
	Anemia^2^
Micronutrient biomarkers	Serum or plasma ferritin; soluble transferrin receptor^104^
	Serum retinol or retinol‐binding protein^1^
	Urinary iodine concentration^104^
	Erythrocyte folate; plasma folate^11^
Dietary intake	Total nutrient intake
	Prevalence of inadequate or excessive intake; proportion of population achieving adequate intake (“effective coverage”)^23^, ^87^
	Proportion of population regularly consuming micronutrient‐rich foods
Process indicators/program monitoring indicators	Proportion of individuals who received an intervention (“reach”), for example, proportion of children 6–59 months of age who received a vitamin A supplement in the previous 6 months^5^
	Proportion of individuals in target group who received intervention (“coverage”)^105^
	Compliance in target group (e.g., proportion of individuals in a target group who consumed at least a certain number of supplements)^104^
	Proportion of the target population that consumes a fortified vehicle (e.g., vitamin A–fortified vegetable oil)^100^
	Proportion of households using adequately iodized salt (“utilization”)^87^, ^104^
	Proportion of food (e.g., wheat flour) samples collected at the market level that are fortified (“access”)
	Regulatory: Functioning national organization tasked with micronutrient deficiency elimination; Legislation for micronutrient programs; Commitment to assess progress of micronutrient programs; Regular monitoring^87^

Note: Examples represent selected common measures, not a comprehensive list.

MN biomarkers can be categorized into markers of exposure, status, function, and effect,^9^ and they vary with respect to the range of MN status that can be detected. Consensus statements on the best biomarkers for the nutrients included in this paper have been published.^10^, ^11^, ^12^, ^13^, ^14^, ^15^, ^16^ Biomarkers of MN excess are available (Table 2), but are generally less well characterized compared to biomarkers of deficiency, particularly in terms of linking these biomarkers with functional or clinical symptoms of toxicity.

**Table 2:** Potential biomarkers of high intake or toxicity for iron, zinc, folic acid, and vitamin A

Micronutrient	Potential biomarkers of excess or toxicity (Reference)	Comments on use
Vitamin A	Retinyl esters^10^	No consensus on cutoffs; affected by fasting status, hypertriglyceridemia, and liver damage
	Retinol isotope dilution^10^	Quantifies liver vitamin A stores, though no consensus on total body stores or hepatic concentrations indicating toxicity; unknown if affected by inflammation
	Breast milk vitamin A^10^	Not homeostatically controlled, so may indicate excessive exposure
Folic acid	Unmetabolized folic acid^11^	No clear relationship between biomarker and clinical adverse effects
Iron	Ferritin^16^	Can indicate high iron stores in the absence of inflammation
	Serum iron, transferrin saturation, and total iron binding capacity (TIBC)^16^	Can indicate high iron stores in the absence of inflammation
	Non‐transferrin‐bound iron (NTBI)^106^	Represents the form of circulating iron likely to cause oxidative damage
Zinc	Plasma copper; ceruloplasmin activity^26^	No clear relationship between biomarker and clinical adverse effects

Given the limited availability of reliable biomarkers of excessive MN status and limited data on existing biomarkers, dietary data are a useful alternative to assess the potential for excessive MN intake and status. Dietary assessment is less invasive than blood sampling, but can still involve complex procedures for data collection and data analysis. Most dietary intake assessment methods are subject to under‐ or overreporting of intake, with consequent implications for the assessment of prevalence of intakes above the tolerable upper intake level (UL).^17^ Individual usual or habitual intake is usually the measure of interest; thus, appropriate methods of data collection and analysis should be used to accurately capture habitual intake.^18^ This may be accomplished using either validated food frequency questionnaires (FFQs) or by combining short‐term assessment methods, such as repeated 24‐h dietary recalls, with appropriate statistical methods to generate population‐level usual intake distributions.^19^, ^20^ The use of appropriate analytical methods is important, because failure to account for intraindividual variation in intake, or use of inappropriate methods to do so, will affect the estimated prevalence of intakes above or below a given threshold.^21^

Different indicators are used to track the delivery of programs and thereby predict their potential impact. Many program evaluations produce estimates of “coverage,” but the specific operational definitions differ. For example, coverage is typically used to refer to receipt of a periodic high‐dose vitamin A supplement (VAS) by a child, where all children 6–59 months of age are considered to be part of the target group,^22^ regardless of their VA status. This target group definition is necessary from a practical perspective since onsite screening for VA deficiency is not feasible. On the other hand, for modeling under the Micronutrient Intervention Modeling (MINIMOD) project, our research group has defined coverage as receipt of an intervention by an individual with biochemically defined MN deficiency or known dietary inadequacy^23^ (Table 3). To avoid confusion, operational definitions should be made clear in the presentation of monitoring results. Through the MINIMOD project, a set of measures of program success and methods for translating the effects of different programs into common units have been developed for selected MNs (Table 3).^23^

**Table 3:** Measures of program impact used by the Micronutrient Intervention Modeling (MINIMOD) project

Indicator	Definition
Reach	Number or percent of individuals who received an intervention^23^
Coverage	Number or percent of individuals who were deficient (defined as biochemical deficiency or individual‐level inadequate intake) and received an intervention^23^
Effective coverage	Number or percent of individuals with inadequate intake who achieved adequate intake as a result of an intervention^23^
Excessive intake	Number or percent of individuals with usual dietary intake above the tolerable upper intake level^74^
Minimum additional intake (MAI)	Number or percent of individuals who received more than a specified threshold of additional nutrient intake from interventions^83^
Lives saved	Number or percent of child lives saved as a result of an intervention^107^
Anemia cases averted	Number or percent of maternal anemia cases averted as a result of an intervention^83^

Selection of the most appropriate measure of program impact will depend on the type of program, the purpose of the assessment, and the time and resources available. For the purpose of identifying excessive intake and predicting program activities that may contribute to excessive intake, the cumulative exposures from both diet and intervention programs are of interest, and thus metrics are needed to assess the combined impacts of both sources of MNs. In our previous work^23^ and in the current review, we rely primarily on dietary assessment methods because the contribution of various programs to daily nutrient intake can be combined together with nutrient intake from diet to estimate and predict total MN exposure. In contrast, it is not straightforward to translate the additional MN received into changes in biomarker concentrations. Dietary assessment can thus be a useful first step toward identifying population groups potentially at risk for excessive intake, and in using predictive modeling to make programmatic decisions that minimize both dietary inadequacy and high or excessive intake. These predictions can then be confirmed with targeted assessments using specific biomarkers of MN status.

While there are numerous advantages to relying on dietary data for the assessment of excessive intake, there are also important limitations to consider, namely difficulties in interpreting the true health implications of MN intake values above the UL. Determining the level of concern that should be attached to observed intakes greater than the UL requires understanding the assumptions and data sources underlying the UL. The information used by the U.S. Institute of Medicine (IOM) to establish ULs is summarized in Table S1 (online only) for selected MNs. The U.S. IOM describes the UL as “the highest usual intake level of a nutrient that poses no risk of adverse effects,”^24^ and the process for setting this value begins with identifying a critical adverse effect upon which to base the UL. For example, the critical adverse effect cited for preformed retinol intake among women of reproductive age is teratogenicity. The literature is then reviewed to identify, preferably, a NOAEL (no observed adverse effects level; the highest level of chronic intake at which no adverse effects have been observed) or, if information is not sufficient to define a NOAEL, a LOAEL (lowest observed adverse effects level; the lowest level of chronic intake at which the selected critical hazard endpoint is observed). The UL is then derived from the LOAEL or NOAEL by applying an uncertainty factor (UF). The UF is a subjective number based on scientific judgment of the committee, taking into account (1) the severity and reversibility of the adverse effect, (2) interindividual variation in susceptibility to adverse effects, and (3) uncertainty due to extrapolation from LOAEL to NOAEL or extrapolation from animal data to humans. FAO and WHO followed a similar approach to develop ULs.^25^

Several important limitations of the UL should be noted in the interpretation of dietary intake in relation to the UL. First, due to the ethical restrictions related to experimental studies designed to induce toxicity, information used to define the UL is typically drawn from observational studies or case reports of individuals with high MN intake. As a result, the quality and quantity of data available for setting the UL are usually limited. For some age groups, particularly children, no information is available to set a UL specific to the age group (Table S1, online only); in such cases, the U.S. IOM extrapolates from other age groups on the basis of metabolic body weight (kg^0.75^).^24^ In addition, the UL typically does not consider issues of bioavailability, and always ignores the potential effects of excessive intake of several MNs simultaneously. For example, the estimated dietary requirements for iron include an assumption about the proportion of iron that is absorbed; in contrast, the UL developed by the U.S. IOM refers to supplemental nonheme iron, without consideration of iron bioavailability.^26^ The UL is also not intended to apply to situations in which individuals are under medical supervision for the treatment of a deficiency. This complicates the application of the UL in cases where interventions are aimed at reducing the prevalence of deficiency rather than simply maintaining adequate MN status. Similarly, the UL is intended for healthy populations and thus its application is unclear in settings with a high burden of infectious disease that may affect nutrient absorption or utilization.

The case of iron illustrates some of the challenges with interpreting intakes above the UL; although there is little evidence of iron intakes exceeding the UL among children (as reviewed below), some studies have suggested risks of providing additional iron to children in amounts below the UL, especially if the children are iron replete. For example, some,^27^, ^28^ but not all,^29^, ^30^, ^31^ trials of iron‐containing micronutrient powder (MNP) distribution to young children have suggested that the use of the powders increased the risk of adverse effects, such as diarrhea and adverse gut microbial profile; these effects have been attributed to the iron content of MNP.^32^ The doses of iron in MNP (typically 10 or 12.5 mg/day) are lower than the UL identified for iron by the U.S. IOM (40 mg/day for children 0–13 years of age^26^), which was based on studies of infants showing no gastrointestinal symptoms following supplementation with iron salts. However, the studies used to develop the UL were mainly from high‐income countries with presumably lower risk of enteric infections and were conducted during 1963–1985 (in the case of U.S. Dietary Reference Intakes), before techniques for assessing gut microbiota were widespread in nutrition and before concerns about adverse interactions between iron supplementation and iron‐dependent pathogens rose to the forefront following the Pemba trial.^33^ A better understanding of the interactions between iron metabolism and infectious disease, as well as impacts on other outcomes, such as child growth and development, is needed to inform an appropriate safe level of iron intake in settings with a high burden of infectious disease.

Two other factors are not limitations per se, but are methodological issues that must be considered in interpreting intakes in relation to the UL. First, for some nutrients, the UL applies only to certain forms or sources of the nutrient.^24^ For example, the UL for folate applies to folic acid present in supplements or fortified foods, but not food folate, and the UL for VA applies only to preformed retinol. Studies that examine the proportion of intakes above the UL but do not differentiate between forms or sources likely overestimate the risk of excessive intakes. Second, the UL is intended to apply to chronic, habitual intake, rather than intake on a single day, so appropriate dietary assessment and analysis methods should be used to capture usual intake distributions.^17^

In summary, analysis of dietary intake and supplement use data remains the most accessible option for determining a population's risk of excessive intake, in the absence of well‐defined biomarkers and/or data on clinical symptoms. The prevalence of intakes above the UL offers an intentionally conservative benchmark for planning programs, but intakes above the UL do not necessarily indicate harm. In populations in which a substantial proportion already exceeds the UL, the physiological risks are unknown; that is, the adverse health effects cannot be quantified with certainty. In such cases, expert review of the type and severity of risk may be used to determine appropriate policy actions. For example, a high prevalence of zinc intakes above the UL has been observed among young children in high‐income countries,^34^, ^35^, ^36^, ^37^ but is generally not considered to be a problem.^38^ On the other hand, excessive intakes of nutrients for which the consequences of excess are more severe could warrant policy changes (e.g., in the case of high preformed retinol intakes among pregnant women). These discussions may be informed by the measurement of biomarkers of excessive MN status, where available.

## Data collection instruments used to determine micronutrients received from programs

Instruments used to assess the amount of MNs received from different dietary sources and intervention programs are summarized in Table 4. These methods have been reviewed previously with respect to generating information for nutrition program planning.^39^, ^40^

**Table 4:** Instruments to assess the theoretical amount of micronutrients received from different dietary sources and intervention programs

Instrument	Focus of data collection	Strengths	Limitations	Example references	Notes on use for examining excessive intakes
24‐h dietary recall	Total food and nutrient intakes on specific days	Captures total dietary intake from all food sources	Time and technical capacity required for collection and analysis of data	74, 108	Must collect multiple days of data and analyze data appropriately to estimate usual intake distributions. Can be combined with a module for supplements and exposure to other programs
Observed weighed diet records	Total food and nutrient intakes on specific days	Same as above, but likely more accurate	Same as above; more time‐consuming data collection		Same as above
Whole diet FFQ (semiquantitative)	Total food and nutrient intakes during specified recall period	Captures usual total nutrient intake, if appropriately designed and calibrated	Formative research needed to define food list and portion sizes		Can be combined with a module for supplements and exposure to other programs
Whole diet FFQ (qualitative)	Patterns of food or nutrient consumption	Captures usual patterns of intake	Does not produce estimates of total intake		Same as above
Micronutrient‐specific or food‐specific FFQ (qualitative or quantitative)	Consumption of specific food or nutrient during specified recall period for selected nutrients consumed in just a few foods	Quick to administer	Does not capture total diet. Formative research needed to define food list	109	Same as above
Fortification Assessment Coverage Tool (FACT)	Consumption of fortified or fortifiable foods; proxy indicators of micronutrient deficiency risk	Relatively quick to administer	Does not capture total diet	100, 105	Same as above
Fortification Rapid Assessment Tool	Consumption of fortifiable foods	Same as above	Same as above	110	Same as above
Household Consumption and Expenditures Surveys	Household apparent consumption of foods	Large sample size and routine collection	Does not provide information on individual food intake; purchased prepared foods often not adequately captured	80, 81, 111	Consider modifications to better capture individual household members’ exposure to intervention programs
Demographic and Health Surveys/Multiple Indicator Cluster Surveys modules	Monitor population health, health services access, and related indicators	Same as above; includes individual exposure to some programs: vitamin A supplements and iron‐folic acid tablets	Very limited data on dietary intake		Consider including indicators related to the coverage of country‐specific programs, including fortification
FAO Food Balance Sheets	Assess availability of food commodities at the national level	Data available annually for most countries	Measures availability rather than consumption; no information on subnational patterns	112, 113	Not appropriate for assessing excessive intake
Dietary diversity score	Brief questionnaire on food groups consumed by household or individual	Rapid; easy to administer; validated against 24‐h recalls as predictor of micronutrient intake adequacy	Does not provide descriptive information on types of foods consumed, or quantitative estimates of intake	114	Provides information on likely adequacy of population micronutrient intake from foods, but not appropriate for assessing excessive intake
Post‐Event Coverage Survey (PECS)	Receipt of high‐dose vitamin A supplement	Rapid; easy to administer	Does not provide information on other interventions, risk factors for deficiency, or dietary intake	115, 116	Consider including indicators related to the coverage of country‐specific programs, including fortification
Fortification Monitoring and Surveillance (FORTIMAS)	Monitoring and surveillance of fortification programs	Relatively low‐resource approach for tracking progress of fortification programs	Data collection focused only on fortification; data not representative	117	Interpret in combination with measures of dietary intake and program exposure

Existing instruments differ with respect to whether they capture information on (1) baseline diets, including self‐prescribed vitamin and mineral supplements; and (2) different types of intervention programs. Twenty four‐hour dietary recall histories and FFQs capture the full diet, but they may not capture usual supplement use (e.g., over 30 days instead of the typical 24‐h recall period) or receipt of other types of MN interventions, unless the questionnaire is specifically adapted to inquire about these sources. Supplemental questionnaires to capture typical supplement use or MN program exposure could be added to 24‐h recall or FFQ data collection with minimal additional time and effort.

Program monitoring tools typically assess reach or coverage of one or more programs but do not measure other sources of MNs in the diet. While the monitoring data are used for tracking program delivery and identifying bottlenecks to implementation, information on diets (and biomarkers of status) is necessary to understand the potential benefits or risks of introduction of a new program. Additional MN intake is unnecessary if diets are already adequate—an extreme example is that of a community in South Africa where liver is commonly consumed and diets contain excessive retinol in the absence of public health programs.^41^ Voluntary fortification, of processed foods in particular, is expanding in many parts of the world.^42^ For example, in the Philippines, over 120 processed food products are fortified with iron, iodine, or VA as part of the Sankap Pinoy Seal program.^43^ In West Africa, fortified bouillon cubes have been introduced on a voluntary basis, although the impact on MN intake is uncertain. Commercial supplements, either purchased voluntarily through markets or prescribed/promoted by physicians, are potentially large contributors to MN intake that should also be considered. Finally, data have emerged highlighting the impact of environmental sources of MNs in the diet that have not traditionally been included in dietary assessment methods, such as mineral concentrations in water^44^ or soil.^45^, ^46^ In affected communities, these sources can contribute substantially to MN intakes, so program planners and researchers should be aware of these cases.

In sum, efforts to assess the risk of excessive MN intake should include information on the contributions from MN intervention programs, as well as sources of MNs in the baseline diet, including fortified processed foods, self‐prescribed supplements, and environmental sources.

## Estimates of micronutrient intake from diet and nutrition programs

We reviewed the existing data to examine (1) the burden of excessive intakes, (2) the distribution of excessive intakes in the population (e.g., among different demographic groups), and (3) the sources contributing to excessive intakes for selected MNs. We searched PubMed for studies reporting excessive MN intake or intake above the UL, and used snowball searching, key contacts, and Google searching to identify reports of national dietary surveys. Many of the existing studies that report the prevalence of intakes above the UL are from high‐income countries; we included these studies because they may provide insights that are relevant to some subgroups in LMICs now or in the future. Because limited data were available from LMICs, we also summarized the results of modeling studies that reported predicted effects of MN intervention programs. Finally, we reanalyzed data from a national dietary survey in Cameroon^47^ to model the predicted effects of various MN program scenarios on the prevalence of inadequate and excessive MN intakes.

We did not undertake a review of the relationship between dietary intake and clinical or biochemical signs of excess; this information has been reviewed in detail by the committees charged with recommending the ULs.^24^, ^25^, ^26^, ^48^ However, we note that these data often come from case reports of individuals with clinical symptoms and small supplementation trials. Fewer data are available from population‐based surveys to suggest whether or not (or at what point) observed intakes above the UL are associated with adverse health effects or to assess the prevalence of potential adverse health effects. The Global Vitamin A Safety Assessment Study (GloVitAS; clinical trials registration no. NCT03030339) is examining this question in relation to VA and is expected to have results by the end of 2019.

### Burden of excessive intakes

#### Vitamin A (retinol)

Retinol intakes above the UL among children have been reported in the United States^34^, ^35^, ^37^ and Canada,^36^ with prevalences ranging from 0% to 4% among children 4–8 years of age^34^ to 59% among children 24–47 months of age.^35^ Among adults, retinol intakes exceeding the UL were uncommon in the United States, Mexico, and several European countries,^49^, ^50^, ^51^, ^52^, ^53^, ^54^, ^55^ but there was some evidence of excessive intake among supplement users.^51^, ^52^ In addition, fortified sugar has been implicated in reports of high VA intake and status in Guatemala,^56^, ^57^ Nicaragua,^58^ and Zambia,^59^, ^60^ although, to our knowledge, national estimates of the prevalence of excessive intake or status are unavailable.

#### Folic acid

In the United States, folic acid intakes above the UL have been reported in preschool children,^34^, ^35^, ^61^ particularly in association with consumption of voluntarily fortified food, such as fortified breakfast cereal: 5–7% of children 1–8 years old in the highest quintile of fortified food intake had folic acid intake above the UL.^34^ Surveys in various countries have suggested that few adults would exceed the UL for folic acid from diet alone,^34^, ^49^, ^50^, ^51^, ^53^, ^54^, ^55^, ^62^ but intakes above the UL were present among ready‐to‐eat cereal (RTEC) consumers in Canada (5.5%^62^) and supplement users in Mexico (12.4%^51^), and 3–6% of individuals in the general U.S. population (≥2 years old and ≥4 years old) would have intakes above the UL when fortification and supplementation are included.^49^, ^50^

#### Iron

In most of the dietary surveys reviewed, the prevalence of iron intake above the UL was low or zero for children^21^, ^35^, ^37^, ^61^, ^63^, ^64^ and adults,^34^, ^49^, ^54^, ^55^, ^62^ with most iron coming from food, including fortified products, such as RTECs. Iron intakes above the UL were observed among supplement users in several countries, ranging from 7% among lactating women in China^65^ and U.S. women^66^ to ∼14% among Hawaiian men^67^ and Mexican women.^51^ In contrast, in Ethiopia, a high prevalence of total iron intake above the UL in the absence of iron supplements or other iron intervention programs was reported among women 15–49 years old (63.6% nationally, ranging from 1.1% in Somali region to 80.2% in Amhara region);^68^ the high iron levels in staple foods have been attributed to extrinsic iron in food (e.g., from soil^69^). In the same survey, children 6–35 months of age in a national sample had a low prevalence of excessive iron intake (<1%),^68^ but in a smaller survey in Ethiopia, 8% of children had total iron intake above the UL from food alone.^70^

#### Zinc

Numerous studies (but not all^64^, ^71^) have reported zinc intakes above the UL, particularly among young children, with many reported prevalences greater than 40%.^21^, ^34^, ^35^, ^36^, ^37^, ^61^, ^64^, ^71^, ^72^ In contrast, among adults, the prevalence of excessive zinc intakes from food appears to be low.^34^, ^49^, ^50^, ^51^, ^53^, ^55^, ^62^, ^65^, ^66^, ^67^ However, prevalences of 7–13% have been observed among adult supplement users in the United States.^50^, ^66^, ^67^

### Simulations of excessive intake among LMICs

Little information is available on the prevalence of excessive intakes in Africa and Southeast Asia, but existing representative survey data suggest that excessive intakes are uncommon in current diets.^68^, ^73^, ^74^, ^75^, ^76^, ^77^, ^78^ In these settings, dietary intake simulations are useful to understand the potential impacts of MN intervention programs on the prevalence of intakes below the EAR and above the UL.

Simulation studies suggest that the predicted impact of large‐scale fortification on both inadequate and excessive intake (i.e., intake above the UL) varies by MN, fortification vehicle and level of fortification, country, subnational region, and target group (e.g., women versus children).^68^, ^74^, ^79^, ^80^, ^81^, ^82^, ^83^ In general, scenarios that included urban areas, children, and fortification of multiple food vehicles were most likely to result in intakes that exceed the UL. Most simulations also identified programmatic combinations that could reduce inadequate intakes without leading to intakes above the UL, suggesting that the results could be useful for planning MN intervention programs. Notably, these simulations are based on the assumption that target nutrient levels in fortified foods are achieved and maintained; limited adherence to program standards will reduce the predicted contributions to both inadequacy and excess.

We analyzed the national dietary survey data from Cameroon^47^ to examine the existing prevalence of dietary adequacy and excess, and the potential effects of intervention programs. In Cameroon, existing fortification programs (addition of 12 mg VA/kg to refined vegetable oil, and addition of 60 mg iron/kg, 95 mg zinc/kg, 5.0 mg folic acid/kg, and 0.04 mg vitamin B12/kg to wheat flour) would not be expected to contribute to excessive intake^74^, ^82^, ^84^ given the reported oil and flour intakes in the population. While fortification at the target levels is predicted to substantially reduce the prevalence of inadequate intake, the effect of oil and flour fortification is limited by the reach (i.e., proportion of the population consuming these foods) in some areas, so we explored scenarios to fortify multiple food vehicles, with varying predicted effects on excessive intake.

Simulations of iron intake among young children under different program scenarios suggest that even the combination of fortified wheat flour (60 mg iron/kg), bouillon cube (600 mg/kg), and supplement distribution (12.5 mg/day for children 12–23.9 months old and 30 mg/day for children 24–59 months old, for 3 consecutive months per year) would not cause intakes to exceed the UL of 40 mg/day for young children. In contrast, fortifying sugar or wheat flour with VA, in addition to oil, was predicted to increase the prevalence of retinol intakes above the UL among young children (17% nationally for sugar fortification at 15 mg/kg and 22% nationally for wheat flour fortification at 8 mg/kg), with particularly large effects in the cities of Yaoundé and Douala, where the consumption of wheat flour and refined vegetable oil was the greatest.^74^ However, fortification of bouillon cube with 48 mg VA/kg, in combination with an oil fortification program, was not predicted to cause excessive intakes (<4% nationally).^74^ Similarly, fortification with folic acid was predicted to result in folic acid intakes above the UL at high levels of fortification of wheat flour, but not bouillon cube.^82^

**Figure 1:**
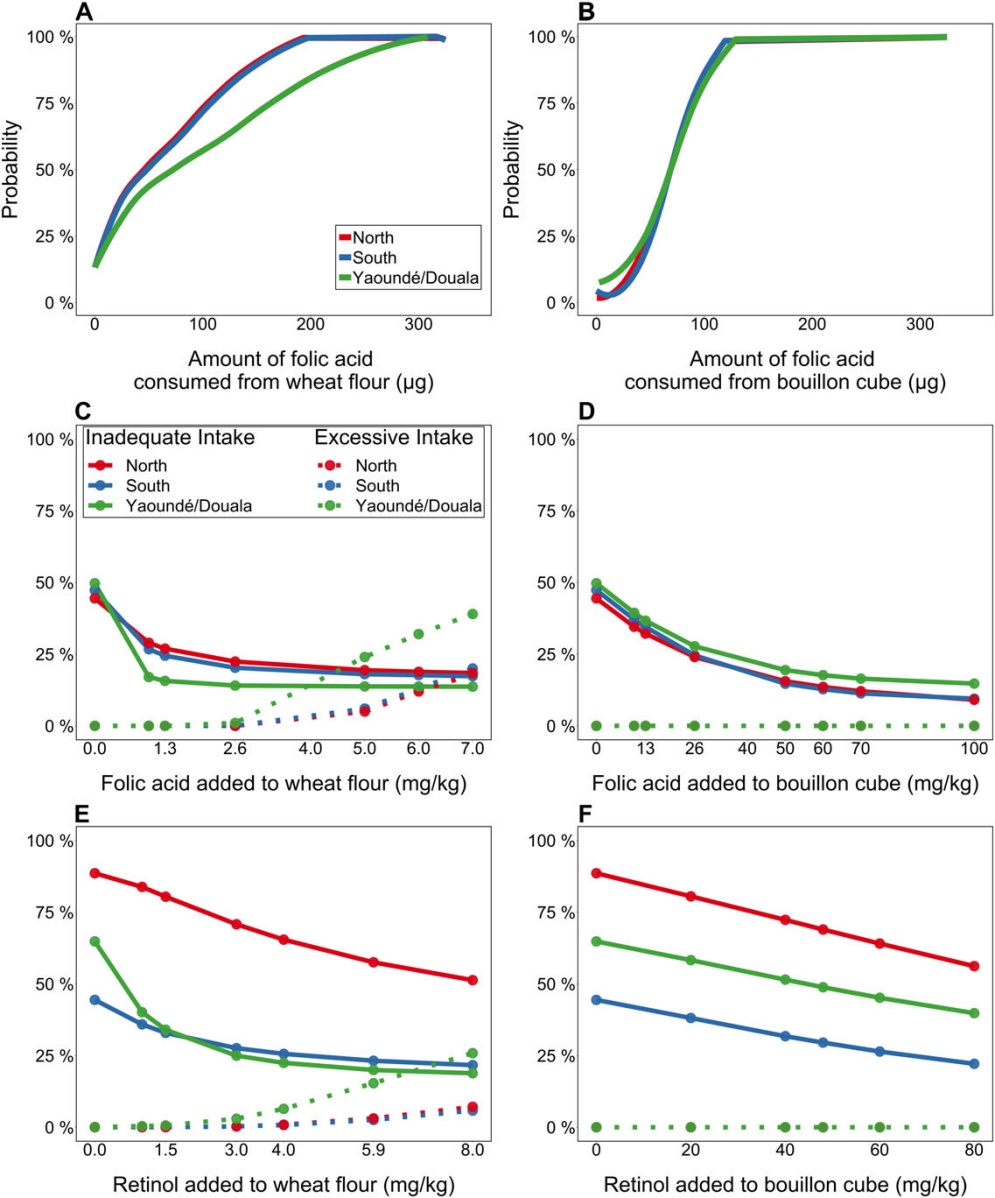
Distribution of folic acid consumption from fortified wheat flour or bouillon cube intake at equivalent fortification levels based on mean national intake, and relative effects of fortification of each vehicle on inadequate and excessive intakes among Cameroonian children 1–4 years of age. For Panels C–F, solid lines indicate prevalence of inadequate intakes, and dotted lines indicate prevalence of intakes above the UL. (A) Cumulative probability distribution of folic acid intake from wheat flour, at 2.6 mg/kg (designed to provide 70 μg/day based on average national intake of 27.0 g/day). (B) Cumulative probability distribution of folic acid intake from bouillon cube, at 70 mg/kg (designed to provide 70 μg/day based on average national intake of 1.0 g/day). (C) Prevalence of inadequate and excessive folate intakes at different levels of fortification of wheat flour. (D) Prevalence of inadequate and excessive folate intakes at different levels of fortification of bouillon cube. (E) Prevalence of inadequate and excessive vitamin A intakes at different levels of fortification of wheat flour. (F) Prevalence of inadequate and excessive vitamin A intakes at different levels of fortification of bouillon cube.

This difference between the effects of wheat flour fortification and bouillon cube fortification on excessive intakes is explained by the distribution of consumption of these two items (Fig. 1; both distributions are estimated using the National Cancer Institute method to remove within‐individual variation^85^, ^86^). The distribution of usual wheat flour consumption is quite skewed: approximately 1/3 of children consumed <5 g of wheat flour on the previous day, whereas consumption at the 95th percentile was more than three times greater than the median consumption. In contrast, the consumption of bouillon cube is more evenly distributed throughout the population (more than 90% of children consumed bouillon cubes on the previous day, and the 95th percentile of consumption was just ∼1.8 times the median consumption) and is similar across macroregions.

In the absence of data on individual dietary intake, fortification levels are often set based on data on average food availability in the population, for example, from sources, such as FAO Food Balance Sheets or industry data. The dietary simulation results from Cameroon suggest that this method may be most useful for foods, such as bouillon cube, that are consumed in consistent amounts among individuals within a particular age group. In contrast, for foods with a skewed intake distribution, such as wheat flour, the average food availability is a less useful proxy for the range of additional daily amounts of MNs potentially delivered through fortification. Thus, where possible, fortification program planning efforts should collect information on the distribution of fortifiable food intake (WHO guidelines recommend collecting information on total intakes of nutrients and fortified foods^87^). Such data can then be used in simulation analyses to identify the appropriate fortification level that will minimize both the prevalence of inadequate intake and the prevalence of intakes above the UL.^74^

### Sources contributing to excessive micronutrient intake and population groups at risk

As reviewed above, data from high‐income countries suggest that risks of excessive intake are mainly present among supplement consumers and, to a lesser extent, individuals with high consumption of fortified foods, such as RTECs. Less is known about voluntary supplement use in LMICs, where government programs may play a larger role than commercial supplements or voluntarily fortified products. The extent to which these programs are likely to contribute to excessive intakes depends on the prevalence of deficiency and the extent to which the supplements can be targeted to individuals at risk of low intake. In the Philippines, pilot data from the GloVitAS project indicated that young children in lower‐middle‐income neighborhoods were commonly given commercial multivitamin‐mineral supplements (RES; unpublished observations). In the United States, where the prevalence of multivitamin‐multimineral supplement use in the previous month was estimated to be 33% in 2003–2006,^88^ supplement users tended to have higher nutrient intakes from food than nonsupplement users,^66^, ^89^ which may place them at even greater risk of excessive intake. However, other studies have reported no difference in nutrient intakes from foods among supplement users and nonusers.^36^, ^51^ Some evidence suggests that fortification of staples may be associated with excessive intakes or status,^57^, ^58^, ^60^ but dietary simulation studies from several countries suggest that excessive intakes are only achieved through fortification of multiple vehicles without adjusting fortification levels to account for this overlap.^74^, ^77^ Similar simulations should be completed in countries with large‐scale fortification programs and available intake data, to describe the potential extent of risks of excessive intake.

The role of new sources of MNs in diets, particularly in processed food products, has received comparatively little attention in LMICs. Products, such as fortified biscuits and instant beverages, are available throughout sub‐Saharan Africa and Southeast Asia, but little information is available on their contribution to MN intakes or their contribution to intake of added sugar and hydrogenated oils. U.S. data suggest that voluntary fortification contributes substantially to MN intakes;^34^ as these products are increasingly available on the global market, they may play an increasing role in MN intakes in LMICs.

Although adult males have been considered an important target group for assessing the risk of excessive intake because their food intake is assumed to be greater than that of other age‐sex groups, the literature reviewed here suggests that excessive intakes of VA, folic acid, and zinc are more common among young children than among adults. Children may be more likely to exceed the UL if they tend to consume more RTECs and supplements compared to adults. However, the high prevalence of intakes above the UL among children may also reflect the fact that for many nutrients, the gap between the EAR and UL is narrower for young children than for adults, reflecting larger UFs used in deriving the UL for children. For example, the UL for VA is twice the EAR for children 1–3 years old, but 3.3 times the EAR for adult men and 4.3 times the EAR for adult women (Table S2, online only). For zinc, the UL is 2.3 times the EAR for children 1–3 years old, but 3.6 times the EAR for adult men and 5 times the EAR for adult women. This uneven EAR–UL gap between age groups may explain why excessive zinc intakes were mainly observed for adults who were supplement users, but excessive zinc intakes among children were not limited to consumers of supplements and fortified foods (as reviewed above).

Few data are available on sociodemographic risk factors for excessive intake. The simulation studies described here suggest that urban populations are at highest risk of excessive intake from staple food fortification in African countries due to greater consumption of industrially processed foods, such as wheat flour, edible oil, and sugar in urban areas. Among adults in the U.S. NHANES survey, males had a higher ratio of intake to UL than did females for iron, zinc, and folic acid,^34^ possibly reflecting greater food intake, since ULs are similar for men and women. In another U.S. study, pregnant women with folic acid intakes above the UL were more likely to be Caucasian or Hispanic, U.S. born, have a higher education, and report no food insecurity or difficulty living on their income.^90^ Similar data on risk factors for excessive intakes are needed from other settings to monitor population subgroups who may be most likely to have high MN intakes.

Taken together, these data suggest that monitoring for excessive intakes should include populations in urban areas, supplement users, and young children, and that assessment methods should include detailed assessment of the use of supplements and fortified foods (whether voluntary or part of a public health program), in addition to MN intake from natural food sources.

## Interpreting program monitoring data and dietary data: weighing benefits and costs

Program managers and policymakers may face situations in which the selection of a particular program will both resolve inadequate intake in some population groups but cause excessive intakes (i.e., intakes above the UL) in other groups. Often a conservative approach is advocated, in which programs are planned and managed such that intakes above the UL are avoided for all groups. For example, the WHO guideline on food fortification states that programs are typically designed such that “predicted probability of inadequate intakes of that specific nutrient is ≤2.5% for population subgroups of concern, while avoiding risk of excessive intakes in other subgroups of the population.”^87^

However, in some cases, it may be reasonable to accept a certain proportion of intakes above the UL if (1) the program is likely to address deficiency; and (2) the total benefits associated with reduced deficiency are deemed to be greater than the risks associated with increases in intake above the UL. Calculating and comparing the consequences of deficiencies with those of intakes above the UL will be challenging for several reasons. First, while some of the physical and cognitive effects of selected MN deficiencies are known for young children, the list is incomplete for all MNs and for other beneficiary groups. Second, little is known about the health or cognitive effects of excessive intake of most MNs, for any beneficiary group. Third, once the effects of deficiencies and excessive intakes are discovered for (at least) selected MNs and beneficiary groups, value weights for these effects will need to be determined to compare the economic and other costs associated with deficiencies with those associated with excessive intake. Identifying these value weights will be especially challenging since (among other things) the subsets of the population suffering from deficiencies (e.g., economically or socially disadvantaged groups) may be very different from those suffering from excessive intake (e.g., more affluent groups), and the onsets over the life cycle and severities of the effects of deficiencies may be different from those associated with excessive intakes.

It is worth noting that the social planner seeking to manage MN intervention programs for addressing deficiencies or excessive intake for a given MN for a given beneficiary group in ways that generate maximal social welfare would seek the set of national and subnational programs that minimized the discounted net costs to society associated with deficiencies *plus* excessive intake, subject to some budget constraint for program implementation. Program choices would be guided by the cost‐effectiveness of specific programs, noting that asymmetries may exist in the cost‐effectiveness of specific policy instruments in reducing MN deficiencies versus excessive intake. For example, removing a MN from a fortified staple food may increase deficiencies while doing little to reduce excessive intake if excessive intakes are explained by other dietary MN sources, such as voluntary supplement consumption by some population subgroups. The complexities of managing such a system increase dramatically when multiple MNs and multiple beneficiary groups are considered. Information on the consequences of deficiency is available for the nutrients considered here, but less information is available on the consequences of intakes greater than the UL, and importantly, the threshold(s) at which toxicity endpoints arise (i.e., the risk curve). Information on economic consequences of deficiency is sparse, and information on economic consequences of excessive intake is almost nonexistent.

Despite these challenges, attempting to quantify and compare the benefits and potential harms of increasing population MN intake is instructive for identifying knowledge gaps for research prioritization. For example, in the case of zinc, there is a substantial evidence base available to quantify the impacts of deficiency on morbidity and mortality among young children.^91^ Analyses for the *Lancet* Nutrition series^92^ estimated that 2.3% of deaths among children under 5 years of age are attributable to zinc deficiency. Disability‐adjusted life years (DALYs) associated with excessive zinc intake could theoretically be calculated if information were available on the (1) prevalence of excessive intake, (2) proportion of individuals with excessive intake who are likely to experience physiological effects of excessive intake at some point in the life cycle (in the case of zinc, the UL is based on altered copper biomarkers, and extrapolated from adults to children), and (3) a measure of the health burden of the effects of excessive intake. There is little evidence to suggest that the observed zinc intakes above the UL are associated with adverse health effects. In two studies of zinc supplementation with doses greater than the UL among young children, no differences in markers of copper status were observed.^93^, ^94^ Even if the dose of zinc were high enough to affect copper metabolism, the resulting clinical and public health burden is unclear. A report from the European Commission Scientific Committee on Food noted that “The 97.5 percentile of total zinc intakes for all age groups are close to the ULs, which, in the view of the Committee, are not a matter of concern.”^38^ Thus, for zinc, the benefits of addressing deficiency appear to outweigh the potential risks, but these calculations are likely to differ by MN, age group, and by geographical and socioeconomic setting.

Through the MINIMOD project, our research team has developed a set of models to predict the nutritional benefits of single or combined MN intervention programs and their respective costs.^23^, ^82^, ^83^, ^95^ These values are combined through an economic optimization model to predict the most cost‐effective set of MN intervention programs over time within subnational regions to meet agreed‐upon targets for program benefits (e.g., minimum number of lives saved), subject to agreed‐upon constraints (e.g., within a maximum budget).^96^ This model has been extended to include a constraint related to excessive intake, such that all program scenarios that result in excessive intakes (intake greater than the UL) above a set threshold will be rejected by the model. Advantages of this approach include: (1) leveraging available data for decision making, (2) offering a transparent framework for considering program benefits and risks, (3) explicit consideration of program costs, and (4) flexibility to propose subnational strategies for addressing MN deficiencies.

Renwick *et al*. described a model for quantifying and comparing the benefits and potential adverse effects of nutrition programs.^97^ The approach draws on the toxicology literature and involves constructing intake—incidence curves for risk of deficiency (or absence of benefit from intervention) and risk of adverse effects associated with excessive intake. The curves represent incidence of a given outcome at different levels of intake (not severity of outcomes according to intake), and are based on (1) existing data linking intake and the selected risks, and (2) assumptions about the variability in susceptibility in the population. These curves are used to construct an optimal range of intake which balances the incidence of each outcome (deficiency and excess). As noted above for the process of selecting a UL, often the endpoints available for defining deficiency or excess are quite different in severity and data availability and quality, and the task of weighing the relative severity of these endpoints is left to the risk manager in this model. Subsequent work introduced the use of DALYs as a common metric for comparing benefits and risks.^98^ The authors illustrated the method using the case of folic acid fortification and compared the benefits (e.g., reduction in neural tube defects, megaloblastic anemia, and colorectal cancer) against the potential risks (e.g., masking vitamin B12 deficiency, colorectal cancer).^98^ Tijhuis *et al*. reviewed the state of benefit–risk assessment in nutrition, including a list of case studies for foods and food components.^99^ While this approach offers a transparent framework for weighting program benefits and risks, a major limitation remains the availability of data to adequately understand the relationships between intake and the incidence and severity of symptoms of deficiency or excess, particularly in the case of excessive intakes, which would not be ethical to induce in a study population.

### Operational challenges of coordinating across programs

Assuming a clear decision could be made about the desirable range of acceptable intakes for a population and the programmatic changes required to achieve this outcome, a practical question is the feasibility of fine‐tuning delivery platforms, programs, and policies to respond to desired changes in program delivery. Ideally, information to guide this process would be available periodically in the form of data on current intake from all sources (including supplements and voluntarily fortified packaged foods) and MN status, MN program coverage among different population subgroups, and quality of program implementation. A full set of information is rarely available; beyond information gaps, other practical challenges arise.

The diverse nutrition interventions available often involve different implementing agencies and different donors; consequently, planning may be siloed and managed by different departments. The choices of interventions are often donor‐ or partner‐driven (particularly in the case of multinational organizations that specialize in delivering specific interventions) and do not consider the contributions from other programs or other types of interventions. Moreover, some sources of MNs may be beyond the reach of policymakers; for example, voluntary food fortification by the private sector may be driven by branding and profitability concerns, and may not be easy for policymakers to influence or manage, depending on the country's policies concerning the regulation of fortification.

To manage national MN program strategies, there is a need for not only a national coordination body that meets regularly, but also for a strong leadership to manage the decision‐making processes in the most desirable manner (e.g., bring a set of competencies together, favor active participation of each, and build consensus). This requires national leadership with a broad vision of nutrition and health to promote programmatic complementarity and synergies and avoid competition among health sector actors. Well‐managed coordination typically depends on individuals who are able to convene multiple stakeholders to focus on a common objective. While this is typically not done on an MN‐specific basis, the concept of coordinating private and public initiatives to promote balanced MN intakes could be integrated into policy and program discussions if the platform exists for regular meetings with a diversity of actors empowered to make decisions.

## Suggested program indicators and data collection mechanisms

### Information needs and tools

Several types of information are necessary to understand the current burden and potential for future risk of excessive MN intakes and to adjust program implementation accordingly. First, information is required on the burden of inadequate and excessive intake and status of the MN of interest, and the population subgroups most likely to have inadequate or excessive intake. Second, data are needed on the types, reach, and performance of available MN programs nationally, subnationally, and among subgroups of interest. These same data can also be used to model improvements to existing programs, and to simulate the effects on benefits and risks of alternative programs, or combinations of them.

Monitoring the reach of programs is necessary to understand the potential for both benefits and risks. Some tools (e.g., the FACT toolkit^100^) have already been developed to assess fortification program reach, and these tools can be modified to fit different applications and settings. Program coverage data may also be collected using special modules that can be added to planned surveys. These modules should include the consumption of voluntarily fortified products and commercial supplement use, as well as exposure to public health programs. Such modules could be included on platforms, such as Demographic and Health Surveys. Involving many stakeholders would be necessary to ensure that all ongoing interventions are considered.

Such monitoring data are critical, but cannot fully substitute for information on total dietary intake, ideally supplemented with biomarker data, for the purpose of assessing benefits and risks. Dietary intake and biomarker surveys are sometimes viewed as prohibitively expensive and time‐consuming; however, research suggests that the benefits of applying this information to the program planning process can outweigh the initial costs.^96^ Additionally, several major efforts are underway to reduce the complexity of collection and analysis of dietary intake data (e.g., the INDDEX project,^101^ FAO/WHO GIFT,^102^ and Intake – Center for Dietary Assessment). Similarly, innovative analytical methods for MN biomarkers could allow for collection of smaller blood volumes, and more field‐friendly matrices, such as dried blood spots, are under development.

In settings where excessive intake is a concern and resources do not permit detailed data collection in the full population, screening tools could be used to identify population subgroups at greatest risk of excess. These groups could then be specifically targeted for detailed assessments of dietary intake and biochemical status, ideally using sensitive markers, such as the retinol isotope dilution method to quantify total body VA stores. This approach was used in the GloVitAS project in the Philippines (NCT 03030339).

Programs should be monitored continuously to track changes in the population status and assess progress toward goals. Dietary and biomarker surveys should be conducted prior to rolling out any major nutrition intervention programs, to provide a baseline against which to evaluate the success of the program, and ideally every 5–10 years thereafter. Program monitoring data should be collected more frequently to provide feedback to program implementers; frequency may range from monthly for routine reporting of programmatic data to every 2–3 years for coverage assessments.

### Weighing risks and benefits

In the absence of detailed data on the risks and hazards of excessive intake, as outlined below, programmatic decisions will require judgment regarding both the relative severity and attendant risks of deficiency and excess. The decision may be straightforward for some nutrients, such as zinc, for which the consequences of intakes above the UL appear to be minimal, and the consequences of deficiency (increased morbidity, mortality, and stunting) are quite severe in low‐income settings. The decision may be more complex for nutrients such as iron when considering the case of children in settings with high burden of diarrheal morbidity, where program managers must weigh the benefits of resolving iron‐deficiency anemia against the potential risk of increased diarrhea incidence. Approaches, such as the MINIMOD model and related frameworks, offer a transparent method for comparing the potential benefits and risks of alternative intervention strategies, and their respective costs.

### Research gaps

Specific research needs to guide the interpretation of data on program exposure and excessive intake include (1) reevaluation of the ULs, including collection of new data on functional outcomes linked to excessive intake; (2) identification and characterization of biomarkers of MN excess, along with thresholds to indicate increasing risk of toxicity; (3) simplification of dietary intake data collection and analysis, with attention to cataloging the nutrient content of supplements^103^ and fortified processed foods; (4) improved tools for estimating the effects of existing and alternative intervention programs on benefits and risks, with special attention paid to intervention program costs; and (5) methods for combining information on deficiency and excess and utilizing this information in policy discussions.

Conceptual, theoretical, and mathematical models are needed to combine the information that will eventually be available on the effects (deficiencies and excessive intake), hazards (value weights for effects multiplied by the effects, such as those reviewed by expert committees in setting the UL), and cost‐effectiveness of programs to address single or multiple MN deficiencies among single or multiple beneficiary groups. Preparing these tools alongside activities to (for example) measure the effects of excessive intake might help establish research priorities. These activities should also include attention to mechanisms for introducing benefit–risk considerations into decision‐making processes.

## Conclusions

The current global situation includes risks of both MN inadequacy and excess, both of which have adverse consequences for health, which may include impacts on mortality and economic productivity. Diets are changing due to planned large‐scale nutrition intervention programs, migration, increases in incomes, and market‐driven forces (e.g., changes in food systems, consequent changes in relative food prices, and voluntary fortification). In general, the few available population‐based surveys from LMICs suggest that current risks of excessive intakes are low for the MNs considered here; however, some exceptions have been noted and dietary simulations suggest that excessive intakes could become more common if multiple programs are implemented without adjusting implementation to account for this overlap. In addition, data from high‐income countries suggest that excessive intakes occur among self‐prescribed supplement users and consumers of large quantities of fortified products (staple foods and/or processed foods). Thus, it is critical to continue to monitor the situation as these sources of MNs become more widely consumed globally. Because excessive intakes are rarely observed from food sources of MNs even in high‐income countries, dietary diversification strategies may be particularly suited to address deficiencies while avoiding excessive intake.

The information that should be collected to monitor nutritional status and program implementation, and to plan and adjust programs accordingly includes (1) information on total dietary intake and MN status, and (2) information on program reach and performance. Many tools are available for monitoring diets and program implementation, but these tools are not always used or used in coordinated ways. Innovations to facilitate collection and analysis of nutritional assessment data, and adaptations of monitoring tools to capture a greater breadth of MN programs and other dietary MN sources will help make the needed information available.

We recommend (1) assessing total dietary intakes and nutritional status for program planning; (2) incorporating rapid screening tools for routine monitoring and surveillance; (3) addressing critical research needs, including evaluations of the current ULs, identifying biomarkers of excess, simplifying dietary data collection, and developing methods for predicting and comparing the risks and benefits associated with alternative MN intervention strategies; and (4) ensuring that monitoring and surveillance data, and the results of simulation modeling, are adequately communicated such that they can inform the decision‐making processes.

## Author contributions

R.E.‐S. conducted the literature review related to the prevalence of excessive intakes and wrote the first draft of the paper. H.L. and R.E.‐S. analyzed the Cameroon dietary survey data. All authors contributed to interpretation of the results and critical review of the manuscript and read and approved the final version.

## Statement

This manuscript was presented at the World Health Organization (WHO) technical consultation “Risk of Excessive Intake of Vitamins and Minerals Delivered Through Public Health Interventions—Current Practices and Case Studies,” convened on October 4–6, 2017, in Panamá City, Panamá. This paper is being published individually but will be consolidated with other manuscripts as a special issue of *Annals of the New York Academy of Sciences*, the coordinators of which were Drs. Maria Nieves Garcia‐Casal and Juan Pablo Peña‐Rosas. The special issue is the responsibility of the editorial staff of *Annals of the New York Academy of Sciences*, who delegated to the coordinators preliminary supervision of both technical conformity to the publishing requirements of *Annals of the New York Academy of Sciences* and general oversight of the scientific merit of each article. The workshop was supported by WHO, the United States Agency for International Development (USAID), and the Bill & Melinda Gates Foundation. The authors alone are responsible for the views expressed in this paper; they do not necessarily represent the views, decisions, or policies of the institutions with which they are affiliated or the decisions, policies, or views of the WHO. The opinions expressed in this publication are those of the authors and are not attributable to the sponsors, publisher, or editorial staff of *Annals of the New York Academy of Sciences*.

## Competing interests

The authors declare no competing interests.

## Supporting information


**Table S1**. Summary of values for the tolerable upper intake level (UL) for vitamin A, folic acid, iron, and zinc set by the U.S. Institute of Medicine.

**Table S2**. Ratio of the U.S. IOM tolerable upper intake level (UL) to the Estimated Average Requirement (EAR) and Recommended Daily Allowance (RDA), by nutrient and population group.
