# Supplementary material for: Weighing the risks of high intakes of selected micronutrients compared with the risks of deficiencies
Source: Ann N Y Acad Sci. 2019 Jun 6;1446(1):81–101. doi: 10.1111/nyas.14128 (PMC6618252; doi:10.1111/nyas.14128)
Supplement: Supplementary file 1 — Table S1. Summary of values for the tolerable upper intake level (UL) for vitamin A, folic acid, iron, and zinc set by the U.S. Institute of Medicine. [file NYAS-1446-81-s001.docx]

**Table S1.** Summary of values for the tolerable upper intake level (UL) for vitamin A, folic acid, iron, and zinc set by the US Institute of Medicine (1, 2).

| **Nutrient** | **Population group** | **Critical adverse effect** | **NOAEL/LOAEL** | **Uncertainty factor** | **UL** |
| --- | --- | --- | --- | --- | --- |
| **Vitamin A**  **(2001)**  **Note:** UL applies to preformed retinol only. | 0-6 mo  7-12 mo | “Hypervitaminosis A” (anorexia, hyperirritability, occipital edema, pronounced craniotabes, bulging fontanels, increased intracranial pressure, skin lesions and desquamation) | LOAEL = 6,460 µg/d (rounded to 6,000) based on averaging lowest doses in 4 case reports (Persson et al, 1965) in children 2.5-5.5 mo. | UF=10 selected to account for extrapolation from LOAEL to NOAEL and for nonsevere and reversible effect (bulging fontanel) and interindividual variability in sensitivity. | 600 µg/d |
|  | 1-3 y | Limited case report data for children and adolescents for hypervitaminosis A (doses ranging from 7000 µg/d in young children to 15,000 µg/d for older children and adolescents) | n/a | Adult UL adjusted based on relative body weight  (given dearth of information and need for conservatism) | 600 µg/d |
|  | 4-8 y | n/a | n/a | Adult UL adjusted based on relative body weight | 900 µg/d |
|  | 9-13 y | n/a | n/a | Adult UL adjusted based on relative body weight | 1,700 µg/d |
|  | 14-18 y, male | n/a | n/a | Adult UL adjusted based on relative body weight | 2,800 µg/d |
|  | 14-18 y female | Teratogenicity of VA intake shortly before or during pregnancy | n/a | Adjusted from value for adult women based on relative body weight | 2,800 µg/d  (same for pregnancy and lactation) |
|  | 19-50 y female | Teratogenicity of VA intake shortly before or during pregnancy | NOAEL = 4,500 µg/d preformed retinol, based on review of studies (mostly case-control, 1 cohort) | 1.5 selected on basis of inter-individual variability in susceptibility, and substantial data on safety of 3,000 µg/d VA supplements. | 3,000 µg/d  (same for pregnancy and lactation) |
|  | >= 19 y, male  >=50 y, female | Liver abnormalities in humans: 1) grossly elevated liver VA levels or hypertrophy of Ito cells, 2) no alcoholism, 3) no hepatitis, 4) no hepatotoxic drug use | LOAEL = 14,000 µg/d based on 2 case studies (1984: 36y old man took 15,000/d for 12 y, 1988: 63-y woman took 14,000/d for 10 y) | UF = 5.0 selected to account for severe, irreversible nature of the adverse effect, extrapolation from LOAEL to NOAEL, and interindividual variation in sensitivity | 3,000 µg/d |
| **Folic acid**  **(1998)** | 0-6 mo  7-12 mo | No data on adverse effects; no concern re: infant’s ability to handle excess | n/a | n/a | Not determinable  “To prevent high levels of intake, the only source of intake for infants should be from food” |
|  | 1-3 y | n/a | n/a | UL for adults was adjusted on the basis of relative body weight and values rounded down | 300 µg/d from fortified foods or supplements |
|  | 4-8 y | n/a | n/a | UL for adults was adjusted on the basis of relative body weight and values rounded down | 400 µg/d from fortified foods or supplements |
|  | 9-13 y | n/a | n/a | UL for adults was adjusted on the basis of relative body weight and values rounded down | 600 µg/d from fortified foods or supplements |
|  | 14-18 y | n/a | n/a | UL for adults was adjusted on the basis of relative body weight and values rounded down | 800 µg/d from fortified foods or supplements |
|  | >= 19 y | Excessive folate intake may precipitate or exacerbate neuropathy in vitamin B-12 deficient individuals | LOAEL = 5 mg/d folate, based on 1) at doses of 5 mg/d and above, more than 100 reported cases of neurological progression. At <5mg/d doses, only 8 well-documented cases. In most cases, folate supplementation maintained the patients in “hematological remission” over a considerable time span. Little info on food folate intake, but most occurred before fortification of breakfast cereal w/folate. | UF = 5, based on severity of neurological complications and use of LOAEL rather than NOAEL. Not larger than 5 due to uncontrolled observation that millsion of people have been exposed to self-treatment with 1/10 the LOAEL (400 µg/d in vitamins) without reported harm. Same UL for elderly and all adults bc 1) devastating and irreversible nature of neurological consequences, 2) data suggesting that pernicious anemia may develop at a younger age in some ethnic groups, 3) uncertainty about occurrence of B12 deficiency in younger age groups | 1,000 µg/d from supplements  (same for pregnant and lactating women) |
| **Iron**  **(2001)** | 0-6 mo  7-12 mo  1-3 y | Adverse GI effects | NOAEL = 40 mg/d based on several studies of Fe supplementation of infants | UF = 1 based on little uncertainty about GI effects | 40 mg/d |
|  | 4-8 y  9-13 y | n/a | Effects of excess supplemental nonheme iron not studied | Adopt value for young children | 40 mg/d |
|  | 14-18 y | n/a | Effects of excess supplemental nonheme iron not studied | Adopt value for adults | 45 mg/d |
|  | >= 19 y | GI effects (including constipation) following supplemental intakes of iron salts | LOAEL = 71 mg/d of supplemental iron salts, based on 97 Swedish men/women taking 60 mg/d as iron fumarate (vs heme+nonheme or placebo). Not possible to derive NOAEL based on GI effects. | 1.5 to account for extrapolation from LOAEL to NOAEL. Higher UF not justified because of self-limiting nature of observed GI effects. | 45 mg/d  (same values for pregnant or lactating women) |
| **Zinc**  **(2001)** | 0-6 mo | Serum copper | NOAEL = 5.8 mg/L formula* milk intake = 0.78 L/d = 4.5 mg/d; no effects on serum copper or cholesterol in 68 term infants suppl with formula with 1.8 or 5.8 mg/L zinc. (Walravens 1976) | Based on duration of study that gave NOAEL (6 mo), used UF = 1.0 and rounded down. | 4 mg/d |
|  | 7-12 mo | n/a | n/a  “No adverse effects of zinc in children and adolescents could be found” | Adjusted from young infants based on relative body weight | 5 mg/d |
|  | 1-3 y | n/a | n/a | Adjusted from young infants based on relative body weight | 7 mg/d |
|  | 4-8 y | n/a | n/a | Adjusted from young infants based on relative body weight | 12 mg/d |
|  | 9-13 y | n/a | n/a | Adjusted from young infants based on relative body weight | 23 mg/d |
|  | 14-18 y | n/a | n/a | Adjusted from young infants based on relative body weight | 34 mg/d  (same for pregnant or lactating) |
|  | 19-50 y  >= 51 y | Effect on copper metabolism (as measured by ESOD activity). Considered, but did not use, data on reduced immune function and reduced HDL cholesterol. | LOAEL – 60 mg/d resulted in lower ESOD activity in 18 women aged 25-40 y. | 1.5, to account for interindividual variability in sensitivity and extrapolation from LOAEL to NOAEL. Higher UF not justified bc reduced copper status is rare. | 40 mg/d  (same for pregnant or lactating) |

References

1. Institute of Medicine (US) Standing Committee on the Scientific Evaluation of Dietary Reference Intakes and its Panel on Folate OBV, and Choline. Dietary reference intakes for thiamin, riboflavin, niacin, vitamin B6, folate, vitamin B12, pantothenic acid, biotin, and choline. Washington, D.C.: National Academies Press (US); 1998.

2. Food and Nutrition Board (FNB) Institute of Medicine (IOM). Dietary Reference Intakes for Vitamin A, Vitamin K, Arsenic, Boron, Chromium, Copper, Iodine, Iron, Manganese, Molybdenum, Nickel, Silicon, Vanadium, and Zinc. Washington D.C.: National Academy Press; 2001.
