# Supplementary material for: Weighing the risks of high intakes of selected micronutrients compared with the risks of deficiencies
Source: Ann N Y Acad Sci. 2019 Jun 6;1446(1):81–101. doi: 10.1111/nyas.14128 (PMC6618252; doi:10.1111/nyas.14128)
Supplement: Supplementary file 2 — Table S2. Ratio of the U.S. IOM tolerable upper intake level (UL) to the Estimated Average Requirement (EAR) and Recommended Daily Allowance (RDA), by nutrient and population group. [file NYAS-1446-81-s002.docx]

**Table S2.** Ratio of the US IOM upper intake level (UL) to the Estimated Average Requirement (EAR) and Recommended Daily Allowance (RDA), by nutrient and population group

|  |  |  | Estimated Average Requirement (EAR) | Recommended Daily Allowance (RDA) | Tolerable upper intake level (UL) | Ratio of UL to EAR | Ratio of UL to RDA |
| --- | --- | --- | --- | --- | --- | --- | --- |
| Retinol ^1^ |  |  |  |  |  |  |  |
|  | Both | 1-3y | 210 | 300 | 600 | 2.9 | 2.0 |
|  | Both | 4-8y | 275 | 400 | 900 | 3.3 | 2.3 |
|  | Female | 9-13y | 420 | 600 | 1700 | 4.0 | 2.8 |
|  | Female | 14-18y | 485 | 700 | 2800 | 5.8 | 4.0 |
|  | Female | 19-50y | 500 | 700 | 3000 | 6.0 | 4.3 |
|  | Female | Female 51+ y | 500 | 700 | 3000 | 6.0 | 4.3 |
|  | Male | 9-13y | 445 | 600 | 1700 | 3.8 | 2.8 |
|  | Male | 14-18y | 630 | 900 | 2800 | 4.4 | 3.1 |
|  | Male | 19-50y | 625 | 900 | 3000 | 4.8 | 3.3 |
|  | Male | 51+ y | 625 | 900 | 3000 | 4.8 | 3.3 |
| Folic acid^2^ |  |  |  | |  |  |  |
|  | Both | 1-3y | 72 ug FA/d = 120 ug DFE | 90 ug FA/d = 150 ug DFE | 300 | 4.2 | 3.3 |
|  | Both | 4-8y | 96 ug FA/d = 160 ug DFE | 120 ug FA/d = 200 ug DFE | 400 | 4.2 | 3.3 |
|  | Female | 9-13y | 150ugFA = 250ugDFE | 180ugFA/d = 300ugDFE | 600 | 4.0 | 3.3 |
|  | Female | 14-18y | 198ugFA/d = 330ugDFE | 240ugFA/d = 400ugDFE | 800 | 4.0 | 3.3 |
|  | Female | 19-50y | 192ugFA/d = 320ugDFE | 240ugFA/d = 400ugDFE | 1000 | 5.2 | 4.2 |
|  | Female | Female 51+ y | 192ugFA/d = 320ugDFE | 240ugFA/d = 400ugDFE | 1000 | 5.2 | 4.2 |
|  | Male | 9-13y | 150ugFA = 250ugDFE | 180ugFA/d = 300ugDFE | 600 | 4.0 | 3.3 |
|  | Male | 14-18y | 198ugFA/d = 330ugDFE | 240ugFA/d = 400ugDFE | 800 | 4.0 | 3.3 |
|  | Male | 19-50y | 192ugFA/d = 320ugDFE | 240ugFA/d = 400ugDFE | 1000 | 5.2 | 4.2 |
|  | Male | 51+ y | 192ugFA/d = 320ugDFE | 240ugFA/d = 400ugDFE | 1000 | 5.2 | 4.2 |
| Iron^3^ |  |  |  | |  |  |  |
|  | Both | 1-3y | 3 | 7 | 40 | 13.3 | 5.7 |
|  | Both | 4-8y | 4.1 | 10 | 40 | 9.8 | 4.0 |
|  | Female | 9-13y | 5.7 | 8 | 40 | 7.0 | 5.0 |
|  | Female | 14-18y | 7.9 | 15 | 45 | 5.7 | 3.0 |
|  | Female | 19-50y | 8.1 | 18 | 45 | 5.6 | 2.5 |
|  | Female | Female 51+ y | 5 | 8 | 45 | 9.0 | 5.6 |
|  | Male | 9-13y | 5.9 | 8 | 40 | 6.8 | 5.0 |
|  | Male | 14-18y | 7.7 | 11 | 45 | 5.8 | 4.1 |
|  | Male | 19-50y | 6 | 8 | 45 | 7.5 | 5.6 |
|  | Male | 51+ y | 6 | 8 | 45 | 7.5 | 5.6 |
| Zinc^4^ |  |  |  | |  | | |
|  | Both | 1-3y | 2.5 | 3 | 7 | 2.8 | 2.3 |
|  | Both | 4-8y | 4 | 5 | 12 | 3.0 | 2.4 |
|  | Female | 9-13y | 7 | 8 | 23 | 3.3 | 2.9 |
|  | Female | 14-18y | 7.3 | 9 | 34 | 4.7 | 3.8 |
|  | Female | 19-50y | 6.8 | 8 | 40 | 5.9 | 5.0 |
|  | Female | Female 51+ y | 6.8 | 8 | 40 | 5.9 | 5.0 |
|  | Male | 9-13y | 7 | 8 | 23 | 3.3 | 2.9 |
|  | Male | 14-18y | 8.5 | 11 | 34 | 4.0 | 3.1 |
|  | Male | 19-50y | 9.4 | 11 | 40 | 4.3 | 3.6 |
|  | Male | 51+ y | 9.4 | 11 | 40 | 4.3 | 3.6 |

^1^Values in µg retinol/d, assuming that all dietary vitamin A is retinol (i.e., ignoring the contribution of provitamin A carotenoids to retinol activity equivalents), for consistency with the tolerable upper intake level, which is based on retinol.

^2^EAR and RDA converted to µg folic acid/d for consistency with UL, ignoring the contribution of dietary folate to dietary folate equivalents.

^3^Values in mg total iron/d, assuming 18% absorption

^4^Values in mg total zinc/d, assuming fractional absorption of zinc of ~41% for men and ~48% for women
